# Mental well-being and sleep quality among vocational college students in Sichuan, China during standardized COVID-19 management measures

**DOI:** 10.3389/fpubh.2024.1387247

**Published:** 2024-05-15

**Authors:** Ru Gao, Hailian Wang, Shan Liu, Xia Wang, Xiaomei Xiong, Si-Yuan Song, Yi Wang

**Affiliations:** ^1^The People's Hospital of Wenjiang, Chengdu, Sichuan, China; ^2^Clinical Immunology Translational Medicine Key Laboratory of Sichuan Province, Sichuan Provincial People’s Hospital, University of Electronic Science and Technology of China, Chengdu, China; ^3^Department of Clinical Medicine, Chengdu University, Chengdu, Sichuan, China; ^4^Baylor College of Medicine, Houston, TX, United States; ^5^Clinical Immunology Translational Medicine Key Laboratory of Sichuan Province, Sichuan Academy of Medical Science and Sichuan, University of Electronic Science and Technology of China, Chengdu, Sichuan, China

**Keywords:** mental health, sleep status, vocational college students, COVID-19 management, family economic status, online behavior, psychological pressure, intervention measures

## Abstract

**Purpose:**

This research investigated the impact of the COVID-19 pandemic on the mental well-being and sleep quality of students in higher vocational colleges in Sichuan, China, identifying key factors influencing their psychological health during this period.

**Methods:**

Between January and February 2022, a comprehensive survey was conducted among students from several higher vocational colleges in Sichuan, utilizing a randomized selection approach to involve 3,300 participants. Data were collected through direct interviews executed by skilled interviewers.

**Results:**

Out of 3,049 valid responses, a significant number reported experiencing symptoms of poor mental health, anxiety, depression, and insomnia, with prevalence rates of 21.2%, 9.7%, 14.1%, and 81.9%, respectively. Factors contributing positively to mental health and sleep included a higher family economic status, reduced stress from the pandemic, and decreased online activity. Conversely, lack of physical activity post-pandemic, disruptions to education and employment, and deteriorating relationships emerged as negative influencers. Interestingly, a lack of pre-pandemic mental health knowledge acted as a protective factor against insomnia.

**Conclusion:**

The ongoing management of COVID-19 has notably influenced the psychological and sleep health of vocational college students, driven by economic, emotional, lifestyle, and educational factors. The findings underscore the necessity for targeted interventions to address these challenges effectively.

## 1 Introduction

As 2022 commenced, COVID-19 emerged with multifaceted transmission pathways, a prolonged asymptomatic phase, robust transmissibility, swift propagation, and a notably elevated mortality rate (1). The pandemic has globally resulted in an extensive toll of infections and fatalities (2). Concurrently, it has exacerbated psychological strain and mental health symptoms across diverse societal sectors (3). Manifestations commonly include depressive and anxious episodes, pervasive worry or fear, sleep disturbances such as insomnia, and a spectrum of somatic symptoms (4–6). Research indicates that anxiety and depression prevalence among infected individuals (31.91, 54.26%) starkly exceeds that in the uninfected demographic (9.18, 21.36%; *p* < 0.05) (7), with the former group also encountering heightened suicidal risks and diminished sleep quality (8).

The human response to crises is multifactorial, influenced by age, educational background, geographical location, economic standing, and the magnitude of the crisis, exemplified by COVID-19 (9, 10). The World Health Organization (WHO) has observed a substantial upsurge in mental disorders post-COVID-19, with anxiety disorders escalating by approximately 25% annually (nearing 100 million cases) and depression by 60 million (11). In China, a substantial survey (56,932 participants) unveiled that during the pandemic, instances of depression, anxiety, insomnia, and acute stress stood at 27.9%, 31.6%, 29.2%, and 24.4%, respectively (12). Participants with confirmed or suspected COVID-19 cases were particularly prone to depressive symptoms (13). The pandemic-induced stress is likely to amplify the prevalence and intensity of anxiety, depression, PTSD, and substance dependency, especially among adolescents globally (14). It underscores the necessity for continued research into the ramifications of the pandemic on youth mental health.

Research across the globe has highlighted the profound impact of the COVID-19 pandemic on college students’ psychological health, with notable increases in anxiety and depression. A comprehensive meta-analysis revealed that anxiety and depression were prevalent in 31 and 34% of college students worldwide, respectively (15). This analysis also noted significant regional variations in these mental health challenges, linking them to the intensity of the local COVID-19 outbreaks. In particular, students from the United States, Spain, and other nations reported escalated mental health issues during the pandemic, marking a rise in anxiety and depression rates from pre-pandemic levels. Similarly, in China, there has been a noticeable uptick in anxiety and depression among college students during the COVID-19 crisis. Research by Nan et al. identified that the rates of depression and anxiety stood at 331.8% and 10.4%, respectively (16). Further studies by Yang Xingjie et al. demonstrated that the prevalence of anxiety and depression symptoms reached 39.0 and 26.9%, respectively (17). These findings underscore the significant psychological toll of the pandemic on the student population, with varying degrees of impact across different countries, influenced heavily by the severity of the COVID-19 situation.

Despite the similarities in the physical and mental development characteristics of university and vocational college students within the broader social context, significant differences exist between higher vocational education and general undergraduate education. These differences are evident in educational philosophy, training objectives, modes of education, and career choices. Particularly in the traditional Chinese context, where academic qualifications hold substantial importance, the lower degree of social recognition for vocational education often leads to feelings of inferiority among vocational college students (18). Amidst the backdrop of higher vocational education, students are at a pivotal stage of psychological and physical maturity. As they navigate social reforms and the rapidly developing market economy, they face escalating academic competition and employment pressures. The accelerated pace of life and reduced communication time further influence their self-evaluation and expectations, frequently leading to discrepancies between their ideals and reality (19). This disparity can result in internal conflicts and significantly challenge their psychological adaptability, increasing their susceptibility to depression, anxiety, PTSD, sleep disorders, and other mental health issues, particularly during the pandemic (20). Notwithstanding the importance of this issue, there remains a scarcity of research addressing mental health concerns, including depression, anxiety, and insomnia, and their contributory elements among vocational college students during the pandemic or its subsequent normalization phase (21). This study is thus focused on exploring the mental health status and sleep quality of higher vocational college students, alongside the determinants that influence these conditions amidst the ongoing normalization of COVID-19 management in Sichuan, China.

The 2020–2023 global COVID-19 pandemic has concluded, offering numerous lessons. During this significant health crisis, the mental health of vocational college students experienced substantial changes, manifesting in fluctuations and negative emotions such as anxiety, discrimination, hypochondria, and emotional collapse (22). Additionally, the preventive and control measures implemented during the pandemic may have heightened the risk of students developing anxiety, depression, and other mental disorders. There were also potential deviations in students’ perceptions of society and events, leading to feelings of dissatisfaction or pessimism, and in some cases, causing social withdrawal or fear of venturing outdoors (23). This study emphasizes that in response to similar future adversities, it is crucial to adopt a proactive approach, leverage positive influences that protect mental health, and ensure the well-being of college students.

## 2 Methods

### 2.1 Research hypotheses

*Hypothesis 1*: The standardized COVID-19 management measures has a negative relationship with Mental Well-Being.

*Hypothesis 2*: Mental Well-Being has a positive relationship on Sleep Quality Among Vocational College Students.

### 2.2 Study population

The research was conducted from January to February 2022 through face-to-face surveys, with strict confidentiality of data maintained. Considering a 12% prevalence of potential mental health issues, such as anxiety or depression, a Type I error probability of less than 0.05, and an allowable error margin of 1%, the minimum sample size was calculated to be 2,800 for this study.

A multi-stage random cluster sampling method was employed. The sampling process consisted of three phases. In the first phase, one district (Wen Jiang) was randomly selected from 12 districts in Chengdu city, with the district serving as the cluster sampling unit. In the second phase, five vocational colleges were randomly chosen from all the vocational colleges in Wen Jiang district, each college acting as a cluster sampling unit. In the final phase, a random selection of 3,300 students was made across these five vocational colleges in Wen Jiang district. The study included students who were actively enrolled, willingly participated, and completed the survey while residing on campus during the study period. We excluded students who were staying at home or were under home quarantine. This study was approved by the ethical committee of Wenjiang District People’s Hospital of Chengdu City (Approval Notice No. 2023-008).

### 2.3 Data collection instruments

This investigation gathered data through a comprehensive questionnaire encompassing demographic details, lifestyle habits, psychological stress, mental health issues, and sleep quality.

### 2.4 Demographics and lifestyle factors

Participants provided demographic data, economic background, parents’ educational levels, and their experiences during the pandemic, including quarantine, stress levels, study habits, physical activity, screen time, job concerns, and interactions with family and friends.

### 2.5 Mental health and sleep assessment tools

The 12-Item General Health Questionnaire (GHQ-12) (24–26), in its Chinese version, was utilized to evaluate the overall mental well-being of individuals over the past month. Recognized for its widespread application in community-based mental disorder epidemiological research, the GHQ-12 includes 12 questions, each with a score ranging from 0 to 1, leading to a maximum score of 12. The scoring system categorizes mental health risk into three tiers: scores of 0 to 1 indicate low risk, scores of 2 to 3 suggest medium risk, and scores of 4 or above denote high risk. A threshold of 3 or higher was employed to identify individuals with potential mental health concerns. In this study, the internal consistency is α = 0.896.

For anxiety assessment, the Generalized Anxiety Disorder-7 (GAD-7) questionnaire was employed (27, 28). This tool, grounded in DSM-IV criteria, aims to measure the severity and impact of seven anxiety symptoms experienced by individuals in community settings over the preceding 2 weeks. The GAD-7 has a scoring system from 0 to 3 for each of its 7 items, totaling a maximum score of 21, with defined levels of anxiety: scores from 0 to 4 indicate no anxiety, scores between 5 and 9 point to mild anxiety, scores from 10 to 14 suggest moderate anxiety, and scores of 15 or above reflect severe anxiety. A score of 10 or more signifies notable anxiety symptoms. In this study, the internal consistency is α = 0.966.

Depressive symptoms were assessed using the Patient Health Questionnaire-9 (PHQ-9) (29, 30), which aligns with DSM-IV criteria for depression. This instrument is designed for the screening of depression in community dwellers over the last 2 weeks, comprising 9 items with scores ranging from 0 to 3, for a total possible score of 27. The scoring breakdown is as follows: 0 to 4 for no depression, 5 to 9 for mild depression, 10 to 14 for moderate depression, and 15 or higher for severe depression. A score of 10 or more is indicative of significant depressive symptoms. In this study, the internal consistency is α = 0.948.

The Insomnia Severity Index (ISI) was used to quantify the severity of recent insomnia symptoms (31). This index includes 7 items, each scored from 0 to 4, culminating in a total score of 28. The severity of insomnia is categorized as follows: 0 to 7 indicates non-significant symptoms, 8 to 14 represents mild insomnia, 15 to 21 denotes moderate insomnia, and 22 to 28 suggests severe insomnia. Scores of 8 or more were considered indicative of insomnia symptoms. In this study, the internal consistency is α = 0.928.

### 2.6 Statistical procedures

Descriptive statistics summarized scale scores and categorical data was expressed in frequencies and percentages. The chi-square test defined as Eq. (1) was used to compare categorical data across groups.


(1) $$\chi^{2}=\sum \frac{{\left(O-E\right)}^{2}}{E}$$

where $\chi^{2}$ is the chi-square test statistic, *O* is the observed frequency, and *E* is the expected frequency under the null hypothesis.

Normally distributed measures were reported as mean ± standard deviation, defined as follows Eq. (2).


(2) $$\mu =\frac{1}{n}\overset{n}{\underset{i=1}{\sum }}x_{i}$$

where μ is the mean of a set of *n* observations *x*_1_, *x*_2_,…, *x_n_*. And the standard deviation of the same set of observations can be calculated as Eq. (3):


(3) $$\sigma =\sqrt{\frac{1}{n}\overset{n}{\underset{i=1}{\sum }}{\left(x_{i}-\mu \right)}^{2}}$$

where σ is the standard deviation.

Binary regression analysis identified factors linked to mental health and sleep disturbances. The logistic function is defined as follows Eq. (4):


(4) $$P\left(Y=1|X\right)=\frac{1}{1+e^{-\left(\beta_{0}+\beta_{1}X_{1}+\ldots +\beta_{n}X_{n}\right)}}$$

where $P\left(Y=1|X\right)$ is the probability of the outcome *Y* being 1 given the predictor variables *X*. $\beta_{n}$ is the regression coefficient. *Xn* is the predictor variable. *e* is the base of the natural logarithm. The regression coefficients were estimated using maximum likelihood estimation. A *p*-value <0.05 was regarded as statistically significant.

## 3 Results

### 3.1 Participant demographics and COVID-19-related factors

#### 3.1.1 Participant demographics

Table 1 delineates the demographic details and COVID-19-related attributes of the study’s participants. The analysis incorporated data from 3,049 individuals, comprising 903 males (29.6%) and 2,146 females (70.4%), with a mean age of 20.9 ± 0.02 years. A majority of respondents, 58.3%, reported living in households of 4 to 6 people. Regarding the previous year’s family economic situation, 75.6% described it as average. The prevalent marital status was single, with 71.9% indicating they were not in a romantic relationship. The educational attainment of parents was varied, with 42.0% of fathers having an education of primary school level or lower, and 40.7% of mothers having completed middle school.

**Table 1:** The demographic of the participants.

**Variables**	***N*(%)**	**Variables**	***N*(%)**
**Gender**		In love	832(27.3)
Male	903(29.6)	Married	25(0.8)
Female	2,146(70.4)	**One-child families**	
**Ethnic**		Yes	832(27.3)
Han	2,725(89.4)	No	2,217(72.7)
Minority	324(10.6)	**Father education level**	
**Total number of families living together**		Primary School or Below	1,281(42.0)
1 to 3 people	1,096(35.9)	Middle school	1,156(37.9)
4 to 6 people	1,718(58.3)	High school or technical secondary school	448(14.7)
More than 7 people	175(5.7)	College degree and above	164(5.4)
**Self-assessment of family economic income last year**		**Mother education level**	
Very good	107(3.5)	Primary School or Below	992(32.5)
Average	2,306(75.6)	Middle school	1,240(40.7)
Poor	636(20.9)	High school or technical secondary school	488(16.0)
**Marital status**		College degree and above	329(10.8)
No love	2,192(71.9)	Severely affected	246(8.1)

#### 3.1.2 The COVID-19 related characteristics of the participants

Table 2 delineates the concerning the COVID-19 pandemic’s impact, 91.6% of students reported they were not quarantined during the epidemic’s normal management phase. About 60.2% felt that their studies had partially resumed to normalcy post-epidemic. Internet usage during the epidemic was moderate among participants, with 56.3% spending between 2 to less than 5 h online daily. Physical activity was notably reduced, with 68.8% not engaging in exercise during the pandemic. The epidemic’s influence on daily routines was significant, yet 64.3% anticipated a gradual return to normal life routines as the situation improved. Prior to the pandemic, 63.9% had sought information on mental health. Relationships with family and friends, including romantic partners, remained unchanged for 69.9% of the participants. Moreover, 64.8% believed that the pandemic had minimally impacted their ongoing education and job search efforts.

**Table 2:** The COVID-19 related characteristics of the participants.

Variables	*N*(%)	Variables	*N*(%)
**You were be quarantined during the normal management of epidemic**		Moderately affected	1,671(54.8)
Yes	256(8.4)	Severely affected	238(7.8)
No	2,793(91.6)	**Whether the rule of the life will gradually return after the epidemic is gradually controlled**	
**The psychological pressure caused by the epidemic and various restrictions**		No recovery	127(4.2)
Little affected	1,499(49.2)	Partial recovery	1,960(64.3)
Moderately affected	1,304(42.8)	Full-Recovery	962(31.6)
Severely affected	246(8.1)	**Learn the mental health related knowledge before the epidemic**	
**Study will return to normal after the epidemic is controlled**		Never really learn	1,100(36.1)
No recovery	86(2.8)	Learn About	1,949(63.9)
Partial recovery	1,836(60.2)	**Relationships with family and friends(including Lover), compared with before of the epidemic**	
Full-Recovery	1,127(37.0)	Little deterioration	70(2.3)
**Average total Internet used per day during normal epidemic management (hours)**		Moderately deterioration	358(11.7)
≤2 h	738(24.2)	No deterioration	1,877(61.6)
2–≤ 5 h	1,718(56.3)	Moderately improvements	325(10.7)
≤5–<7 h	484(15.9)	Great improvement	92(3.0)
7 h≤	109(3.6)	Unclear	223(7.3)
**Physical exercise during the epidemic**		**Epidemic affected the continuing education and job hunting**	
Yes	950(31.2)	Little affected	1,977(64.8)
No	2,099(68.8)	Moderately affected	6,601(21.6)
**Eepidemic affected the rule of the life**		Severely affected	152(5.0)
Little affected	1,140(37.4)	Unclear	260(8.5)

### 3.2 General health and mental well-being during COVID-19

According to Table 2, the assessment of the general health, anxiety, depression, and sleep quality revealed that 645 participants (21.2%) displayed general health concerns (GHQ-12 scores ≥ 3), 295 individuals (9.7%) exhibited anxiety symptoms (GAD-7 scores ≥ 10), 430 students (14.1%) showed signs of depression (PHQ-9 scores ≥ 10), and a significant 2,497 (81.9%) experienced insomnia (ISI scores ≥ 8) in the context of the pandemic (Table 3).

**Table 3:** Single factor analysis of the detection rate of mental health disorder, sleep status in college students (*n* = 3,049).

Variables	GHQ-12 ≥ 3分	X^2^	GAD-7 ≥ 10分	X^2^	PHQ-9 ≥ 10分	X^2^	ISI ≥ 8	X^2^
**Gender**								
Male	175(19.4)	2.42	95(10.5)	1.05	145(16.1)	4.05	695(77.0)	21.03*^##^*
Female	470(21.9)		200(9.3)		285(13.3)		1,802(84.0)	
**Ethnic**								
Han	580(21.3)	0.26	267(9.8)	0.44	383(14.1)	0.049	2,233(81.9)	0.04
Minority	65(20.1)		28(8.6)		47(14.5)		264(81.5)	
**Total number of families living together**								
1 to 3 people	217(19.8)	5.18	106(9.7)	2.27	167(15.2)	3.42	875(79.8)	5.13
4 to 6 people	399(22.4)		174(9.8)		245(13.8)		1,479(83.2)	
More than 7 people	29(16.6)		15(8.6)		18(14.3)		143(81.7)	
**Self-assessment of family economic income last year**								
Very good	13(12.1)	34.71*^##^*	11(10.3)	13.94*^#^*	16(15.0)	28.59*^##^*	69(64.5)	22.7*^##^*
Average	446(19.3)		198(8.6)		283(12.3)		1,901(82.4)	
Poor	186(29.2)		86(13.5)		131(20.6)		527(82.9)	
**Marital status**								
No love	455(20.8)	1.33	203(9.3)	4.06	299(13.6)	1.85	1,800(82.1)	0.29
In love	186(22.4)		87(10.5)		126(15.1)		677(81.4)	
Married	4(16.0)		5(20.0)		5(20.0)		20(80.0)	
**One-child families**								
Yes	161(80.6)	2.23	82(9.8)	0.043	124(14.9)	0.11	653(78.5)	8.98*^#^*
No	1,733(78.2)		213(9.6)		306(13.8)		1,844(83.2)	
**Father education level**								
Primary School or Below	274(21.4)	0.83	110(8.6)	9.42*^#^*	159(12.4)	7.52	1,049(81.9)	0.26
Middle school	239(20.7)		118(10.2)		169(14.6)		955(82.6)	
High school or technical secondary school	100(22.3)		41(9.2)		71(15.8)		368(82.1)	
College degree and above	32(19.5)		26(15.9)		31(18.9)		125(76.2)	
**Mother education level**								
Primary School or Below	208(21.0)	0.36	90(9.1)	4.43	125(12.6)	4.35	808(81.5)	1.44
Middle school	259(20.9)		120(9.7)		175(14.1)		1,023(82.5)	
High school or technical secondary school	108(22.1)		43(8.8)		75(15.4)		403(82.6)	
College degree and above	70(21.3)		42(12.8)		55(16.7)		263(79.9)	
**You were be quarantined during the normal management of epidemic**								
Yes	71(27.7)	7.25*^#^*	28(10.9)	0.51*^#^*	45(17.6)	2.79	225(87.9)	6.77*^#^*
No	574(20.6)		267(9.6)		385(13.8)		2,272(81.3)	
**The psychological pressure caused by the epidemic and various restrictions**								
Little affected	260(17.3)	46.61*^##^*	98(6.5)	67.04*^##^*	161(10.7)	69.15*^##^*	1,135(75.7)	75.94*^##^*
Moderately affected	297(22.8)		141(10.8)		194(14.9)		1,145(87.8)	
Severely affected	88(35.8)		56(22.8)		75(30.5)		217(88.2)	
**Study will return to normal after the epidemic is controlled**								
No recovery	29(33.7)	21.64*^##^*	19(22.1)	23.53*^##^*	27(31.4)	30.64*^##^*	71(82.6)	34.50*^##^*
Partial recovery	421(22.9)		193(10.5)		277(15.1)		1,563(85.1)	
Full-recovery	195(17.3)		83(7.4)		126(11.2)		863(76.6)	
**Average total Internet access per day during normal epidemic management (hours)**								
≤2 h	124(19.2)	23.36*^##^*	63(21.4)	17.72*^#^*	82(19.1)	34.76*^##^*	551(22.1)	36.78*^##^*
2– ≤ 5 h	363(56.3)		166(56.3)		242(56.3)		1,435(57.5)	
≤5–<7 h	121(18.8)		43(14.6)		71(16.5)		415(16.6)	
7 h≤	37(5.7)		23(7.8)		35(8.1)		96(3.8)	
**Physical exercise during the epidemic**								
Yes	171(18.0)	8.23*^#^*	81(8.5)	2.89	102(10.7)	12.91*^##^*	722(76.0)	32.35*^##^*
No	474(22.6)		214(10.2)		328(15.6)		1,775(84.6)	
**Eepidemic affected the rule of the life**								
Little affected	188(16.5)	65.35*^##^*	87(7.6)	37.93*^##^*	120(10.5)	65.99*^##^*	849(74.5)	68.64*^##^*
Moderately affected	362(21.7)		159(9.5)		237(14.2)		1,437(86.0)	
Severely affected	95(39.9)		49(20.6)		73(30.7)		211(88.7)	
**Whether the rule of the life will gradually return after the epidemic is gradually controlled**								
No recovery	41(32.3)	27.71*^##^*		29.12*^##^*	35(27.6)	33.78*^##^*	99(78.0)	47.49*^##^*
Partial recovery	449(22.9)		27(21.3)		298(15.2)		1,675(85.5)	
Full-recovery	155(16.1)		202(10.3)		97(10.1)		723(75.2)	
**Learn the mental health related knowledge before the epidemic**								
Never really learn	253(23.0)	3.51	105(9.5)	0.03	161(14.6)	0.40	874(79.5)	6.92 *^#^*
Learn about	392(20.1)		190(9.7)		269(13.8)		1,623(83.3)	
**Relationships with family and friends(including Lover), compared with before of the epidemic**								
Little deterioration	25(35.7)	21.58*^#^*	15(21.4)	26.88*^##^*	20(28.6)	26.06*^##^*	57(81.4)	8.12
Moderately deterioration	89(24.9)		52(14.5)		59(16.5)		298(83.2)	
No deterioration	353(18.8)		151(8.0)		227(12.1)		1,544(82.3)	
Moderately improvements	109(23.6)		48(10.4)		71(15.4)		383(82.9)	
Great improvement	26(25.0)		10(9.6)		18(17.3)		76(73.1)	
Unclear	43(24.2)		19(10.7)		35(19.7)		139(78.1)	
**Epidemic affected the continuing education and job hunting**								
Little affected	366(18.5)	46.5 *^##^*	170(8.6)	18.64*^##^*	241(12.2)	32.46*^##^*	1,595(80.7)	15.52*^#^*
Moderately affected	168(25.5)		78(11.8)		114(17.3)		574(87.0)	
Severely affected	60(39.5)		27(17.8)		41(27.0)		124(81.6)	
Unclear	51(19.6)		20(7.7)		34(13.1)		204(78.5)	

^#^*P* < 0.05, ^##^*P* < 0.001.

### 3.3 Associations with general health and mental well-being

Participants from lower economic backgrounds, with quarantine history, facing psychological stress, spending 2–5 h daily online, lacking physical activity, and having poor familial and social relationships reported poorer general health (*p* < 0.05, or *p* < 0.001; Table 2). Those who perceived their studies and daily routines as heavily impacted by COVID-19, along with those whose educational pursuits and job searches were disrupted post-COVID-19, also reported poorer health (*p* < 0.001).

### 3.4 Anxiety and related factors

Anxiety was more prevalent among students from lower-income families, those with fathers holding college degrees or higher, individuals with quarantine experiences, subjects under high psychological stress, those spending 2–5 h online daily, and those with weaker ties to family and partners. The pandemic’s disruption to daily life, education, and job prospects was also linked to increased anxiety (Table 3).

### 3.5 Depression correlates

Depression correlated with factors such as lower economic status, high psychological stress, quarantine history, disrupted studies, 2–5 h of internet use, lack of exercise, strained relationships, and pandemic-related impacts on daily life, education, and employment (*p* < 0.05; Table 3).

### 3.6 Insomnia indicators

Insomnia was associated with lower economic status, not being an only child, quarantine experience, higher stress levels, disrupted studies, 2–5 h of daily internet use, absence of physical activity, lack of prior mental health knowledge, and the pandemic’s influence on daily routines, educational continuation, and job hunting (*p* < 0.05; Table 3).

### 3.7 Analysis of predictive factors for general health and anxiety symptoms during the pandemic

#### 3.7.1 Influences on general health status

Table 4 delineates the associations between self-perceived family economic status and general health. Participants who rated their family’s economic status as either ‘very good’ (OR = 0.362, 95%CI = 0.19–0.69, *P* < 0.005) or ‘average’ (OR = 0.655, 95%CI = 0.53–0.81, *P* < 0.001) the previous year were found to have a considerably reduced risk of a higher GHQ score. Similarly, those who reported minimal (OR = 0.554, 95%CI = 0.40–0.77, *P* < 0.001) or moderate (OR = 0.65, 95%CI = 0.48–0.88, *P <* 0.05) psychological pressure due to the pandemic and related restrictions also exhibited a lower risk of elevated GHQ scores. Reduced total daily online time was associated with lower GHQ scores, with ≤2 h (OR = 0.50, 95% CI =0.31–0.80, *P* < 0.05) and 2–≤ 5 h (OR = 0.61, 95% CI = 0.39–0.96, *P* < 0.05) being significant. Participants who experienced little (OR = 0.47, 95%CI = 0.34–0.60, *P* < 0.001) or moderate (OR = 0.0.60, 95%CI = 0.44–0.82, *P* < 0.005) disruption to their daily routines due to the pandemic were less likely to have higher GHQ scores. However, those who reported severe impacts on their education and job searching activities (OR = 1.85, 95%CI = 1.11–3.09, *P* < 0.001) had a higher risk of an increased GHQ score.

**Table 4:** Binary logistics regression analysis of mental health, sleep status in college students (*n* = 3,049).

Variables	*β*	*S_E_.*	*P*	*OR(95% CI)*	Variables	*β*	*S_E_.*	*P*	*OR(95% CI)*
**Impact factors of the mental health**					The relationship with family and friends (including lovers) had Little deterioration	1.25	0.37	0.001	3.50(1.70–7.17)
Self-assessment of family income was very good last year	−1.02	0.33	0.002	0.36(0.19–0.69)	The relationship with family and friends (including lovers) had great improvement	1.22	0.47	0.010	3.38(1.35–8.47)
Self-assessment of family income was average last year	−0.42	0.11	0.000	0.65(0.53–0.81)	**Impact factors of the depression symptoms**				
The psychological pressure caused by the epidemic and various restrictions was Little affected	−0.59	0.16	0.000	0.55(0.40–0.77)	Self-assessment of family income was average last year	−0.46	0.13	0.000	0.63(0.49–0.81)
The psychological pressure caused by the epidemic and various restrictions was Moderately affected	−0.43	0.16	0.006	0.65(0.48–0.88)	The psychological pressure caused by the epidemic and various restrictions was Little affected	−0.92	0.18	0.000	0.40(0.28–0.56)
Average total Internet access per day was ≤2 h during normal epidemic management	−0.70	0.24	0.004	0.50(0.31–0.80)	The psychological pressure caused by the epidemic and various restrictions was Moderately affected	−0.67	0.17	0.000	0.51(0.37–0.72)
Average total Internet access per day was 2–≤ 5 h during normal epidemic management	−0.49	0.23	0.032	0.61(0.39–0.96)	Average total Internet used was ≤2 h per day, during normal epidemic management	−1.07	0.26	0.000	0.34(0.21–0.57)
Eepidemic Little affected rule of the life	−0.76	0.17	0.000	0.47(0.34–0.65)	Average total Internet used was 2–≤ 5 h per day hours during normal epidemic management	−0.85	0.24	0.000	0.43(0.27–0.68)
Eepidemic moderate affected rule of the life	−0.51	0.16	0.001	0.60(0.44–0.82)	Average total Internet used was ≤5–<7 h per day during normal epidemic management	−0.89	0.26	0.001	0.41(0.25–0.69)
Continuing education and job hunting	0.62	0.26	0.018	1.85(1.11–3.09)	Had the Physical exercise during the epidemic	−0.32	0.13	0.018	0.73(0.56–0.95)
**Impact factors of the anxiety symptoms**					The relationship with family and friends (including lovers) had great improvement	1.0	0.37	0.008	2.71(1.30–5.65)
Self-assessment of family income was average last year	−0.31	0.15	0.040	0.73(0.55–0.99)	**Impact factors of the insomnia symptoms**				
The psychological pressure caused by the epidemic and various restrictions was Little affected	−1.08	0.20	0.000	0.34(0.23–0.50)	Female	−0.25	0.11	0.023	0.78(0.63–0.97)
The psychological pressure caused by the epidemic and various restrictions was Moderately affected	−0.63	0.19	0.001	0.53(0.37–0.77)	Self-assessment of family income was very good last year	−0.67	0.25	0.008	0.52(0.32–0.84)
Average total Internet access per day was ≤2 h during normal epidemic management	−0.85	0.29	0.004	0.43(0.24–0.76)	Be quarantined during the normal management of epidemic	0.47	0.21	0.023	1.60(1.07–2.40)
Average total Internet access per day was 2–≤ 5 h during normal epidemic management	−0.74	0.27	0.007	0.48(0.28–0.82)	The psychological pressure caused by the epidemic and various restrictions was Little affected	−0.60	0.22	0.007	0.55(0.36–0.85)
Average total Internet access per day was ≤5–<7 h during normal epidemic management	−0.91	0.31	0.003	0.40(0.22–0.73)	Study partial return to normal after the epidemic is controlled	0.23	0.11	0.045	1.25(1.01–1.56)
Eepidemic little affected the rule of the life	−0.61	0.22	0.005	0.54(0.35–0.83)	Average total Internet used was ≤2 h per day, during normal epidemic management	−0.97	0.32	0.003	0.38(0.20–0.71)
Epidemic Moderately affected the rule of the life	−0.50	0.20	0.013	0.61(0.41–0.90)	Eepidemic little affected the rule of the life	−0.63	0.23	0.007	0.54(0.34–0.84)
The relationship with family and friends (including lovers) had Moderately deterioration	1.25	0.44	0.004	3.49(1.49–8.78)	Before the epidemic had Learned about the mental health related knowledge	−0.23	0.10	0.027	0.79(0.65–0.98)

#### 3.7.2 Predictors of anxiety

Table 4 further demonstrates that participants with an ‘average’ economic assessment last year (OR = 0.73, 95%CI = 0.55–0.99, *p* < 0.05) had a reduced likelihood of elevated GAD-7 scores. Individuals experiencing little (OR = 0.34, 95%CI = 0.23–0.50, *p* < 0.001) or moderate (OR = 0.53, 95%CI = 0.37–0.77, *p* < 0.05) psychological pressure from the pandemic and restrictions also showed a lower risk of increased GAD-7 scores. Daily online time of ≤2 h (OR = 0.43, 95%CI = 0.24–0.76, *p* < 0.005), 2–≤ 5 h (OR = 0.48, 95%CI = 0.28–0.82, *p* < 0.005), and 5–<7 h (OR = 0.40, 95%CI = 0.22–0.73, *p* < 0.05) were linked to a higher risk of increased GAD-7 scores. Those with a minimal (OR = 0.54, 95%CI = 0.35–0.83, *p* < 0.05) or moderate (OR = 0.61, 95%CI = 0.41–0.90, *p* < 0.05) pandemic impact during standard management were more likely to have heightened GAD-7 scores. A deterioration in the relationship with family and friends (including lovers) that was slight (OR = 3.50, 95%CI = 1.70–7.17, *p* < 0.005) or significant (OR = 3.38, 95%CI = 1.35–8.47, *p* < 0.05) corresponded to a greater risk of increased GAD-7 scores, as did a moderate deterioration (OR = 3.49, 95%CI = 1.49–8.78, *p* < 0.005).

#### 3.7.3 Predictive factors for depression

As illustrated in Table 4, students who perceived their family’s economic standing as average in the previous year exhibited a reduced likelihood of heightened PHQ-9 scores (OR = 0.63, 95%CI = 0.49–0.81, *p* < 0.001). Those minimally (OR = 0.40, 95%CI = 0.28–0.56, *p* < 0.001) or moderately (OR = 0.51, 95% CI = 0.37–0.72, *p* < 0.001) affected by pandemic-induced psychological pressure were found to have an increased risk of higher PHQ-9 scores. Daily internet use for ≤2 h (OR = 0.34, 95%CI = 0.21–0.57, p < 0.001), 2–≤ 5 h (OR = 0.43, 95%CI = 0.27–0.68, *p* < 0.001), and 5–<7 h (OR = 0.41, 95%CI = 0.25–0.69, *p* < 0.005) were associated with an elevated risk of depression symptoms. Engaging in physical exercise during the pandemic was linked to a lower risk of increased PHQ-9 scores (OR = 0.73, 95%CI = 0.56–0.95, *p* < 0.05). An improved relationship with family and friends, including partners, was correlated with a higher risk of depression symptoms (OR = 2.71, 95%CI = 1.30–5.65, *p* = 0.008).

#### 3.7.4 Influences on sleep quality

Table 4 also identifies factors influencing sleep quality. Females had a lower risk of experiencing increased ISI scores (OR = 0.78,95%CI = 0.63–0.97, *p* < 0.05). Participants with a ‘very good’ self-assessed family income from the previous year were less likely to have elevated ISI scores (OR = 0.52, 95% CI = 0.32–0.84, *p* < 0.05). Minimal psychological pressure due to the pandemic was associated with a lower risk of poor sleep quality (OR = 0.55,95%CI = 0.36–0.85, *p* < 0.05). Those who utilized the internet for ≤2 h daily had a reduced risk of sleep issues (OR = 0.38, 95%CI = 0.20–0.71, *p* < 0.005). A small impact of the pandemic on daily life was also linked to better sleep quality (OR = 0.54,95%CI = 0.34–0.84, *p* < 0.05). Pre-pandemic knowledge about mental health correlated with better sleep status (OR = 0.54, 95%CI = 0.65–0.98, *p* < 0.05). Being quarantined (OR = 1.60, 95%CI = 1.07–2.40, *p* < 0.05) and a partial return to normal studies post-pandemic (OR = 1.25, 95%CI = 1.01–1.56, *p* < 0.005) were associated with an increased risk of insomnia.

#### 3.7.5 Interrelationships among mental health variables

As depicted in Table 5, Pearson correlation analysis revealed significant relationships between anxiety and depression (R = 0.861, *p* < 0.001), as well as between anxiety, depression, and insomnia (R = 0.644, 0.716, *p* < 0.001), indicating a strong interconnectivity among these mental health issues.

**Table 5:** Analysis of the correlation between mental health status and sleep status in college students (*n* = 3,049).

	GHQ-12 total scores	GAD-7 total scores	PHQ-9 total scores	ISI total scores
GHQ-12 total scores	1			
GAD-7 total scores	0.399^**^	1		
PHQ-9 total scores	0.390^**^	0.861^**^	1	
ISI total scores	0.309^**^	0.644^**^	0.716^**^	1

^**^P < 0.001. GHQ-12,12-Item General Health Questionnaire; GAD-7, Generalized Anxiety Disorde-7; PHQ-9, Patient Health Questionnaire-9; ISI, Insomnia Severity Index.

## 4 Discussion

This study focuses on examining the mental and sleep health of vocational college students in Sichuan, China, during the ongoing COVID-19 pandemic’s normalization phase, identifying key factors influencing their psychological well-being. Our research underscores the significant effects various determinants have on students’ mental health and sleep patterns, advocating for customized psychosocial strategies to improve their overall well-being. In educating students through the pandemic, acknowledging the substantial stress and potential psychological impact infectious disease outbreaks can exert is crucial. Experiencing negative emotions during such times is natural and should not lead to excessive mental strain. Acceptance of these feelings is vital for timely mental adjustment and proactive engagement with the pandemic. Rational processing of epidemic information, understanding the virus’s nature, staying informed about the pandemic without succumbing to misinformation, and maintaining trust in governmental public health measures can transform anxiety into constructive action. Ensuring personal protection without undue worry, maintaining open communication channels, and leveraging technology for social interactions can mitigate feelings of isolation. Upholding a healthy lifestyle, including adhering to regular eating and sleeping schedules, helps sustain normalcy. Additionally, establishing sound hygiene practices and mastering stress management and emotional release techniques are essential for psychological resilience (32).

### 4.1 The prevalence of general health, anxiety, depression, and insomnia in vocational college students

Our research reveals that in the context of normalized COVID-19 management, the prevalence of general health, anxiety, depression, and insomnia in vocational college students in Sichuan stands at 21.2%, 9.7%, 14.1%, and 81.9%, respectively. These figures are notably higher than those reported in earlier studies, where anxiety and depression rates among 1,676 college students were documented at 9.4% and 27.7% (33, 34). It is evident that the pandemic has exacerbated the mental health challenges faced by college students (35). Possible reasons include significant psychological stress and mental health symptoms experienced by students during the pandemic. Constraints such as home quarantine, limited social interaction, disrupted routines, employment concerns, and strained family relations could contribute to heightened anxiety, depression, and insomnia (36, 37). Nonetheless, some studies suggest that students’ mental health during the normalized phase may be better than at the onset of the pandemic, which could be attributed to post-traumatic growth (38). Additionally, variations in the timing of research, regional and cultural distinctions among study subjects, or the employment of diverse research methodologies could account for discrepancies in the findings.

### 4.2 Family economic conditions, psychological stress, and online engagement affected the mental health and sleep status during the pandemic

The current study also found that students with more robust family economic conditions, lower psychological stress, and reduced online engagement during the pandemic had better overall health and fewer symptoms related to anxiety, depression, and insomnia. The pandemic’s normalization phase has seen a significant impact on the financial status of many students’ families (39). A more stable economic background may help students manage the emotional toll of the pandemic, reducing psychological stress and symptoms of anxiety, depression, and insomnia, aligning with the findings of this study (40). In line with previous studies, our analysis also reveals that students subjected to quarantine measures and those not participating in physical activities tended to report higher levels of mental health challenges, including symptoms of anxiety, depression, and insomnia. Engaging in regular physical activity and maintaining a structured routine can bolster students’ resilience and willpower, enhance physical capabilities, enrich emotional wellbeing, boost self-efficacy and achievement, thereby augmenting their psychological capital. Such practices lead to a more persistent and stable sense of contentment. This study underscores the positive correlation between physical activity, a regulated lifestyle, and the psychological capital of college students, emphasizing the importance of these factors in fostering mental health (41).

### 4.3 Physical activity and a consistent lifestyle, engaging in exercise affected the mental health disorder, sleep status during the pandemic

This research indicates a positive association between physical activity and a consistent lifestyle with enhanced mental health outcomes among college students during the pandemic’s normalization phase, notably in reducing symptoms of depression and insomnia. Engaging in exercise is posited to strengthen the resilience and psychological health of college students throughout and subsequent to the pandemic (39). It has been previously documented that the occurrence of anxiety and insomnia symptoms saw an uptick preceding and during the normalized phase of the pandemic (42). Further, existing literature supports that anxiety and depression can negatively impact sleep quality, while poor sleep may aggravate symptoms of depression and anxiety, a finding that aligns with the outcomes of this study. In essence, physical activities such as jogging, yoga, tennis, and swimming have been identified as beneficial for alleviating anxiety. The critical benefit of exercise lies in its capacity to facilitate comprehensive muscle relaxation, which is particularly significant for managing anxiety disorders. Furthermore, physical exercise is known to enhance the secretion of neurotransmitters like dopamine, norepinephrine, and serotonin, contributing to a reduction in symptoms of anxiety and depression. Additionally, engaging in regular physical activity can diminish the physiological stress response, thereby lowering muscle tension and heart rate, which, in turn, helps alleviate anxiety symptoms. Exercise also fosters social engagement and enhances social support, further contributing to the mitigation of anxiety symptoms (43, 44).

### 4.4 Epidemic affected the continuing education and job hunting

The necessity for further investigation into this critical domain is evident. Our findings reveal that students facing disruptions in their educational and career trajectories tend to suffer from deteriorated mental health, a conclusion that aligns with prior studies (45, 46). The adverse effects of the pandemic on internships and job opportunities (47), along with its sustained global economic repercussions, have posed significant challenges for businesses and intensified competition in the job market for graduates. The broadening scale of university admissions has placed higher vocational college students at a disadvantage, particularly in comparison to their undergraduate and graduate counterparts, in terms of academic capabilities and employment prospects.

There exists a subconscious drive among these students to continuously enhance their skills and never allow complacency, spurred by the heightened employment pressures catalyzed by the pandemic’s economic fallout in recent years. This accumulation of stressors can escalate physical and mental strain, potentially leading to severe health issues and psychological disturbances in extreme cases(?). Highlighting the importance of supporting students in their academic and professional endeavors as a means to promote mental well-being is crucial (48, 49).

### 4.5 Relationships with family and friends (including lover) affected by the epidemic

Our research reveals a decline in the quality of students’ relationships with family, peers, and romantic partners since the onset of the pandemic, correlating with an increase in anxiety and depression levels amid COVID-19’s normalized management. These findings echo prior studies that have documented elevated anxiety and depression rates among college students during these challenging times (37). The family unit serves as a critical emotional anchor throughout an individual’s development, significantly influencing college students’ mental health. Evidence suggests that strong familial bonds can mitigate feelings of depression and loneliness, fostering mental well-being among students. The support, empathy, and warmth from family members are pivotal for the psychological health of students, especially given their physical distance from home during college years (50). The role of family in shaping students’ personalities and mental health education is profound (38). Peer interactions constitute a vital aspect of the college experience, significantly impacting students’ mental health. Positive peer relationships can provide a sense of security, compensating for deficiencies in family and academic environments (51). Rust and support from peers can enhance students’ self-esteem and social skills, decreasing the likelihood of depression and anxiety. Conversely, poor peer relations can lead to negative mental health outcomes, including feelings of rejection, isolation, and even suicidal ideation. Enhancing social skills and fostering genuine friendships can improve the quality of peer relationships, which is beneficial for mental health maintenance. Romantic relationships are notably significant during college years, with the satisfaction derived from these relationships closely linked to students’ mental health. Research indicates that students in satisfying relationships experience higher levels of happiness and lower incidences of negative emotions like depression and anxiety (52). Furthermore, engaging in romantic relationships can bolster social competencies and self-awareness, while inspiring personal goals and aspirations. Nevertheless, relationship challenges or breakups can exert considerable psychological stress and detriment to students’ well-being.

### 4.6 Mental health knowledge and self-regulation skills affect the mental health and sleep quality

Our findings intriguingly suggest that those lacking prior knowledge of mental health were less prone to suffer from insomnia. This observation implies that individuals aware of mental health issues before the pandemic may have been more susceptible to its stressors, increasing their risk of insomnia. It has been documented that the emotional state of college students regarding sleep has shifted significantly due to the pandemic. The introduction of online learning and the pressures associated with isolation have potentially deteriorated their sleep quality and emotional well-being. Furthermore, possessing knowledge about mental health could allow for some degree of self-regulation and adjustment psychologically. Additionally, college students who consistently engage in physical activity display greater resilience in managing public health crises. Research indicates that students dedicating 30 min to 1 h daily to exercise experience the least stress. Regular moderate exercise is an effective strategy to mitigate stress and anxiety, with physical activity fostering positive mental states. This, in turn, supports mental health, alleviates anxiety, and promotes sound sleep (53). Earlier studies reveal that students experiencing higher stress, fatigue, and sleepiness shortly after the onset of the COVID-19 pandemic reported elevated anxiety and depression levels, alongside compromised sleep quality, in response to public health emergencies. Hence, it is crucial to focus on the capacity of vocational college students to navigate public health crises through the acquisition of mental health knowledge and self-regulation skills, aiming to reduce anxiety and depression and enhance sleep quality (54). The importance of continued research in this area is evident.

### 4.7 The characteristics of the vocational students and the contributions of the study for psychological construction in college students

This study highlights that as societal dynamics evolve, the perspectives of college students in our country have also undergone significant transformations. Universities can only enhance the relevance of mental health education for college students by comprehensively understanding and addressing their mental states, which are continuously evolving. Higher vocational college students, who are particularly curious about external information, often experience psychological fluctuations in response to public opinion and crises. Consequently, it is crucial to prioritize mental health education for vocational college students during public crises to foster their overall development (55). For future educational initiatives, the following strategies are recommended: First, enhance the awareness of mental health education among teachers, stressing the integration of mental health education into vocational training programs, with teacher evaluations in higher vocational colleges including an assessment of their awareness of mental health education. Second, enrich the appeal of mental health education activities in higher vocational colleges by ensuring that these activities are engaging, varied, and capable of stimulating student participation. Third, emphasize the central role of students by valuing their involvement and fostering their enthusiasm for participating in activities, tailoring mental health education strategies to the unique physical and mental development needs of vocational students, especially during public crises. Fourth, leverage the supportive role of network information technology in education, utilizing tools such as WeChat public accounts for diverse instructional delivery, to enhance learning interest and effectively focus on the core aspects of mental health education for higher vocational college students, thereby promoting their mental well-being.

It is important to acknowledge the limitations of this study. Firstly, the cross-sectional nature of this survey means that the relationships between mental health issues, insomnia, and potential risk factors cannot be inferred as causational. Secondly, the reliance on self-reported data for general health, anxiety, depression, and sleep status may not fully correspond with evaluations conducted by mental health professionals. Thirdly, due to varying social conditions, the applicability of our findings to other regions may be limited.

## 5 Conclusion

It is necessary to infiltrate mental health education in daily teaching, create a good environment and atmosphere of mental health education, and implement mental health education in order to maintain the normalization and sustainable development of mental health education. Teachers should integrate more contemporary content into mental health education based on the psychological state of vocational college students, take some hot issues in the new era as mental health education resources, so as to arouse students’ thinking, and realize the goal of mental health education in the discussion and reflection of psychological problems, so as to seek the effectiveness of education. Actively excavate the educational resources contained in public crisis events, guide students to maintain a good attitude toward people in the face of danger, and face difficulties bravely.

The COVID-19 pandemic has significantly altered individuals’ lifestyles and behaviors, leaving a lasting impact on society. Among the affected, college students have faced considerable psychological stresses, necessitating a comprehensive approach to their mental well-being. It is essential for governmental bodies, educational institutions, community organizations, and families to work in concert toward enhancing the mental health framework for students. This collaborative effort should include leveraging public resources like mental health platforms and support hotlines established by governments, alongside colleges and universities initiating mental health screenings and offering a mix of virtual and in-person mental health seminars. These steps ensure students facing mental health issues receive prompt, professional psychological counseling or treatment to help stabilize their emotional state. Moreover, community and familial support structures need to actively provide accessible measures, with parents particularly encouraged to engage more deeply in communication with their college-aged children, fostering a supportive environment for their mental health. Conducting this study 2 years after the pandemic’s onset has shed light on the ongoing mental health and sleep challenges faced by students in Sichuan, China, during this global health emergency. The findings underscore the critical need for targeted health policies and psychosocial interventions designed to support student mental health resilience in the face of public health crises.

## Data availability statement

The original contributions presented in the study are included in the article/supplementary material, further inquiries can be directed to the corresponding authors.

## Ethics statement

This study was approved by the Ethical Committee of Wenjiang District People’s Hospital of Chengdu City (Approval Notice No. 2023-008). The studies were conducted in accordance with the local legislation and institutional requirements. Written informed consent for participation was not required from the participants or the participants’ legal guardians/next of kin in accordance with the national legislation and institutional requirements.

## Author contributions

RG: Data curation, Writing – original draft, Funding acquisition, Validation. HW: Data curation, Writing – original draft. SL: Formal analysis, Methodology, Writing – original draft. XW: Formal analysis, Writing – original draft. XX: Formal analysis, Writing – original draft. S-YS: Conceptualization, Investigation, Validation, Writing – review & editing. YW: Conceptualization, Funding acquisition, Investigation, Project administration, Supervision, Writing – review & editing.
